# Implementation and use of technology-enabled blood pressure monitoring and teleconsultation in Singapore’s primary care: a qualitative evaluation using the socio-technical systems approach

**DOI:** 10.1186/s12875-023-02014-8

**Published:** 2023-03-16

**Authors:** Sok Huang Teo, Evelyn Ai Ling Chew, David Wei Liang Ng, Wern Ee Tang, Gerald Choon Huat Koh, Valerie Hui Ying Teo

**Affiliations:** 1 National Healthcare Group Polyclinics, Singapore, Singapore; 2 Ministry of Health Office for Healthcare Transformation, Singapore, Singapore; 3 Saw Swee Hock School of Public Health, National University of Singapore, Singapore, Singapore

**Keywords:** Health information technology, Socio-technical systems, Implementation, Primary health care hypertension, Blood pressure monitoring

## Abstract

**Background:**

Telemedicine is becoming integral in primary care hypertension management, and is associated with improved blood pressure control, self-management and cost-effectiveness. This study explored the experiences of patients and healthcare professionals and their perceived barriers and facilitators in implementing and using a technology-enabled blood pressure monitoring intervention with teleconsultation in the Singapore primary care setting.

**Methods:**

This was a qualitative study embedded within the Primary Technology-Enhanced Care Hypertension pilot trial. Patients were selected purposively and invited to participate by telephone; healthcare professionals involved in the trial were invited to participate by email. Individual semi-structured interviews were conducted in English or Mandarin with thirteen patients and eight healthcare professionals. Each interview was audio-recorded and transcribed verbatim. Data were analyzed inductively to identify emergent themes which were then grouped into the dimensions of the socio-technical systems model to study the interactions between the technical, individual and organizational factors involved in the process.

**Results:**

Several emergent themes were identified. The factors involved in the implementation and use of the intervention are complex and interdependent. Patients and healthcare professionals liked the convenience resulting from the intervention and saw an improvement in the patient-provider relationship. Patients appreciated that the intervention helped form a habit of regular blood pressure monitoring, improved their self-management, and provided reassurance that they were being monitored by the care team. Healthcare professionals found that the intervention helped to manage workload by freeing up time for other urgent matters. Nevertheless, participants highlighted challenges with usability of the equipment and management portal, data access, and some expressed technology anxiety. Participants suggested patient segmentation for the intervention to be more targeted, wished for a more user-friendly equipment and proposed allocating more resources to the intervention.

**Conclusions:**

The implementation and use of telemedicine for hypertension management can engender various benefits and challenges to patients, healthcare professionals and the healthcare system. Stakeholder feedback gathered on the sociotechnical aspects of the technology should be taken into consideration to guide the design, implementation and evaluation of future telemedicine interventions in primary care.

**Trial registration:**

This study was registered on ClinicalTrials.gov on October 9, 2018. ID: NCT03698890.

**Supplementary Information:**

The online version contains supplementary material available at 10.1186/s12875-023-02014-8.

## Background

Hypertension is one of the most prevalent chronic diseases in Singapore, affecting 35.5% of adults aged between 18 and 74 years old [1]. It is commonly managed in primary care in Singapore and globally [2]. In the Global Burden of Disease Study 2019, elevated blood pressure (BP) was one of the top five risks of attributable deaths and a key risk factor for ischemic heart disease and stroke, which were the leading causes of disability-adjusted life years of people aged 50 years and above [3, 4].

Besides lifestyle modifications and pharmacological treatment if indicated, telemedicine has increasingly been considered as an integral part of hypertension management in primary care to improve access to services, quality of care, productivity and prevention of cardiovascular diseases [5]. Telemedicine refers to the systematic provision of healthcare services remotely via information and communications technology, and includes tele-collaboration, tele-treatment, tele-monitoring and tele-support in Singapore [6]. It has been used in the management of chronic diseases in primary care, such as asthma, diabetes, hypertension and multimorbidity, to improve disease control and quality of life [7–11].

The most well-received telemedicine application for hypertension management is BP telemonitoring, which allows BP readings to be transmitted remotely from the patient’s home to the physician [12]. Studies have shown that BP telemonitoring, along with telecounseling and management by a team of healthcare professionals (HCPs), is associated with reductions in BP, healthcare utilization, mortality and cost, and improvements in patient self-management, empowerment, quality of life and patient-provider relationship [13–15]. The Telemonitoring and Self Management in the Control of Hypertension (TASMINH2) and the Telemonitoring and Self-management in Hypertension (TASMINH4) trials in the UK [16–18] and the Scale-Up BP study in Scotland [19] showed that BP telemonitoring is feasible in primary care, facilitates better medication management and well-received by patients and HCPs, who are important stakeholders in its implementation. Nevertheless, a multitude of cultural practices, technical, individual and organizational factors which differ between countries can influence the safe and effective implementation and use of telemedicine.

Singapore is a multi-ethnic and urbanized country in Asia, with a small population size of 5.7 million people and high mobile phone and internet penetration rates [20, 21]. Like many developed countries, Singapore is facing the challenges posed by an ageing population, increasing chronic disease prevalence, and rising healthcare cost. Primary healthcare in Singapore comprises unsubsidized private clinics and subsidized public institutions (polyclinics), with the latter managing most chronic disease patients. Telemedicine was already in use for chronic disease management in primary care before the COVID-19 pandemic in the form of nurse-based tele-support services [22]. The pandemic has propelled its use to unprecedented levels, as it enabled the continuation of chronic disease management while adhering to safe distancing measures.

The Primary Technology-Enhanced Care – Hypertension (PTEC-HT) pilot trial showed that there is an opportunity for telemedicine to improve clinical outcomes and cost-effectiveness of chronic disease management programs in Singapore’s primary care. PTEC-HT was a quasi-experimental trial conducted in a polyclinic in central Singapore with patients recruited from September 2018 to March 2019. The study methodology and main results have been reported elsewhere [23]. 217 patients with hypertension (office BP ≥ 140/90 mmHg) or hypertension with hyperlipidemia were recruited to the intervention or usual care group and followed up for 6 months. All patients were cared for by healthcare teams called teamlets, each comprising two family physicians, a care manager who is a nurse trained in chronic disease management, and a care coordinator who is a lay person that follows up with patients on their appointments. Each participant in the intervention group was loaned a Bluetooth-enabled home BP monitor and mobile data network connecting gateway device, and was asked to monitor BP at least once weekly. The BP readings were automatically uploaded to a web-based portal for clinical management. Patients with well-controlled BP were reviewed regularly through telephone consultations with the care managers (scheduled teleconsultations). Care managers would contact patients if unexpectedly high readings were detected to check on their condition (unscheduled teleconsultations). Medications were adjusted over the phone (medication titration) if the physicians deemed it clinically necessary. The intervention of BP telemonitoring combined with teleconsultation was found to have improved BP control and patient satisfaction, and was cost-effective.

The current qualitative study, embedded within the PTEC-HT trial, explored the experiences and perspectives of patients and HCPs on the barriers and facilitators to the implementation and use of the intervention in the Singapore primary care setting.

## Methods

### Recruitment of interview participants

To capture a range of experiences of implementing and using the intervention, the research team identified HCPs who were involved in the implementation and patients who had remained in the trial to participate in the current study. Twenty patients were selected purposively and invited by telephone, and a maximum variation sample was sought based on age and sex characteristics. Ten HCPs were identified and invited via email. Due to the scope of the ethical approval, patients who withdrew from the trial could not be recruited into the current qualitative study.

### Data collection

Individual semi-structured interviews were chosen to elicit participant thoughts and feelings about the implementation of the BP telemonitoring intervention [24]. Interview questions were developed based on the study aims, literature review and discussions within the research team. The topic guide (Additional file 1) was pilot-tested and covered experiences and opinions on using the telemonitoring equipment, BP self-monitoring, teleconsultation, medication adjustments and lifestyle modifications. Additional probes were used to clarify and discover in-depth information. Data collection and analysis were carried out concurrently. The topic guide was refined iteratively as needed, including adding prompts and questions, based on emerging findings from preceding interviews.

All participants provided written consent for this study. The interviews were conducted face-to-face with each participant between March and September 2019, each interview lasted 20–60 minutes. Two investigators (SHT and EALC) who are trained in qualitative methods conducted the interviews in English or Mandarin in an enclosed room in the polyclinic. Each interview was audio-recorded and transcribed verbatim. Repeat interviews were not carried out. Transcripts were anonymized to ensure data confidentiality.

Ethical approval was received from the National Healthcare Group Domain Specific Review Board.

### Analysis

Each transcript was checked for accuracy against the recording. Data analysis was conducted following the framework method [25].

Two investigators (SHT and EALC) read and re-read the transcripts independently to familiarize with the data and independently coded the data. SHT is a research fellow with a master’s degree in public health and EALC is a research fellow with PhD in Communications. Data were analyzed inductively to examine the perspectives of different participants, highlighting similarities and differences, and generating unanticipated insights [26]. Data gathered from patients and HCPs were analyzed separately. A reflexivity journal was maintained by the investigators to record their views before the analysis. Initial codes were discussed and a working analytical framework was agreed upon. Constant comparison was employed to ensure consistency in coding. Differences in opinion were resolved through consensus on the main themes and subthemes that emerged from the data. An Excel spreadsheet was used to generate a matrix to chart the data with the cases in rows, codes in columns and summarized data in the cells. After coding all transcripts, relationships among categories were explored to raise the analytical level from categorical to thematic. Thematic data saturation was reached after the 21^st^ interview of both patients and HCPs, as no new themes emerged from the data by then. The different codes, categories, and themes were grouped into a coding tree chart that illustrated the patient and provider perspectives on the BP telemonitoring and teleconsultation intervention.

**Fig. 1:**
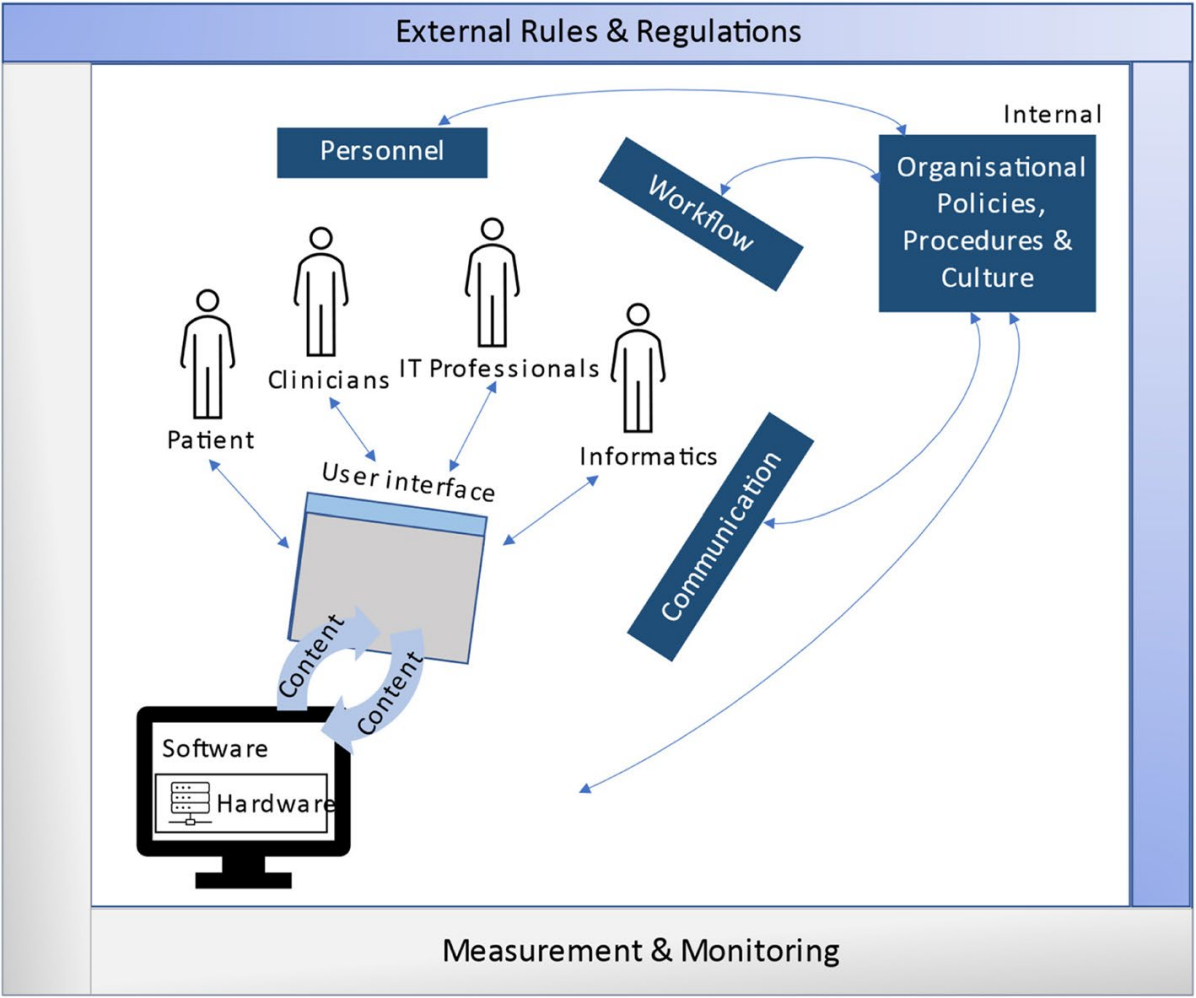
Socio-technical systems (STS) model by Sittig and Singh

The themes were further mapped to the dimensions of the socio-technical systems (STS) model to understand the relationships between the sociotechnical aspects of the intervention [26]. The STS model (Fig. 1) was developed by Sittig and Singh to comprehensively study the interconnectedness between the key factors that influence the success of health information technology interventions within complex adaptive healthcare systems like primary care. It consists of eight dimensions [27, 28]:

- (1) Hardware and software – This dimension focuses on the technical aspects required to run the applications, including the physical equipment and software.
- (2) Clinical content – This dimension considers the data collected that are useful for patients and HCPs to manage patient care and inform clinical decisions.
- (3) Human-computer interface – This dimension includes aspects of the system that users can see, touch or hear as they interact with the application.
- (4) People – This dimension represents the patients and HCPs who are involved in the implementation and use of the intervention, and how the intervention helps them think and makes them feel.
- (5) Workflow and communication – This dimension focuses on the interaction between the patients and HCPs to accomplish patient care as well as the impact of the intervention on the HCP workflows.
- (6) Internal organizational policies, procedures, and culture – This dimension can affect every other dimension in the model, as organizational policies and procedures can affect the availability of hardware and software and clinical workflows related to the intervention.
- (7) External rules and regulations – This dimension focuses on the external regulations and priorities that can facilitate or impede the implementation and use of the intervention.
- (8) System measurement and monitoring – This dimension focuses on measuring and monitoring the availability, usage, effectiveness and outcomes of the intervention regularly.

The latter two dimensions were omitted from this study as the analysis did not find any relevance of the data to these dimensions.

NVivo 12 (QSR International Pty Ltd) was used for data management and analysis. This study is reported according to the Consolidated criteria for Reporting Qualitative research (COREQ) checklist [29].

## Results

Thirteen patients and eight HCPs were enrolled into the current study. Participant characteristics are shown in Tables 1 and 2 for patients and HCPs, respectively. 61.5% of patients interviewed were males and mean age was 55.7 years (range from 35 to 73 years). Most patients were Chinese, attained pre-university or tertiary education level and working full-time. We interviewed two family physicians, one nurse clinician, three care managers and two care coordinators. Most HCPs were females and the number of years in practice ranged from 1.5 to more than 40 years.

**Table 1:** Participant characteristics: Patients (*N* = 13)

Characteristic	n (%)
**Gender**	
Male	8 (61.5)
**Ethnicity**	
Chinese	12 (92.3)
Malay	1 (7.7)
**Highest education level**	
Primary	1 (7.7)
Secondary	3 (23.1)
Pre-University	2 (15.4)
Tertiary	7 (53.8)
**Employment status**	
Working part-time	2 (15.4)
Working full-time	9 (69.2)
Retired	2 (15.4)

**Table 2:** Participant characteristics: Staff (*N* = 8)

Characteristic	n (%)
**Gender**	
Female	7 (87.5)
**Ethnicity**	
Chinese	8 (100.0)
**Profession**	
Family physician—Approved medication titration; provided clinical input	2 (25.0)
Nurse clinician—Supported backend coordination of implementation of intervention	1 (12.5)
Care manager—Performed teleconsultation and monitoring of BP readings	3 (37.5)
Care coordinator—Trained participants to use remote BP monitor; provided follow-up technical support	2 (25.0)
**Years in practice**	
1–4	2 (25.0)
5–9	1 (12.5)
10–14	2 (25.0)
15–19	1 (12.5)
20 and above	2 (25.0)

**Fig. 2:**
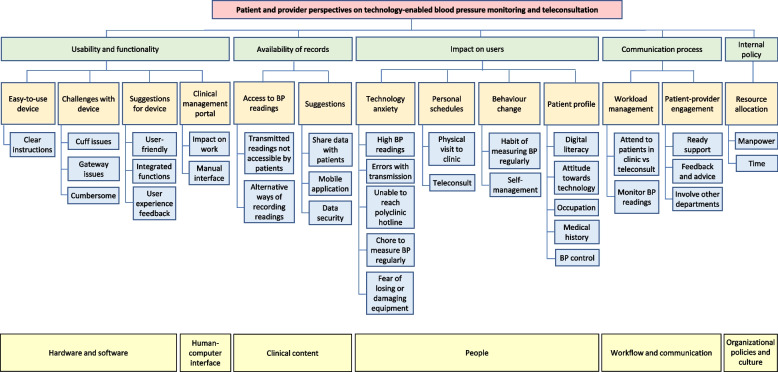
Coding tree of patient and provider perspectives on the intervention, mapped to the STS dimensions

**Fig. 3:**
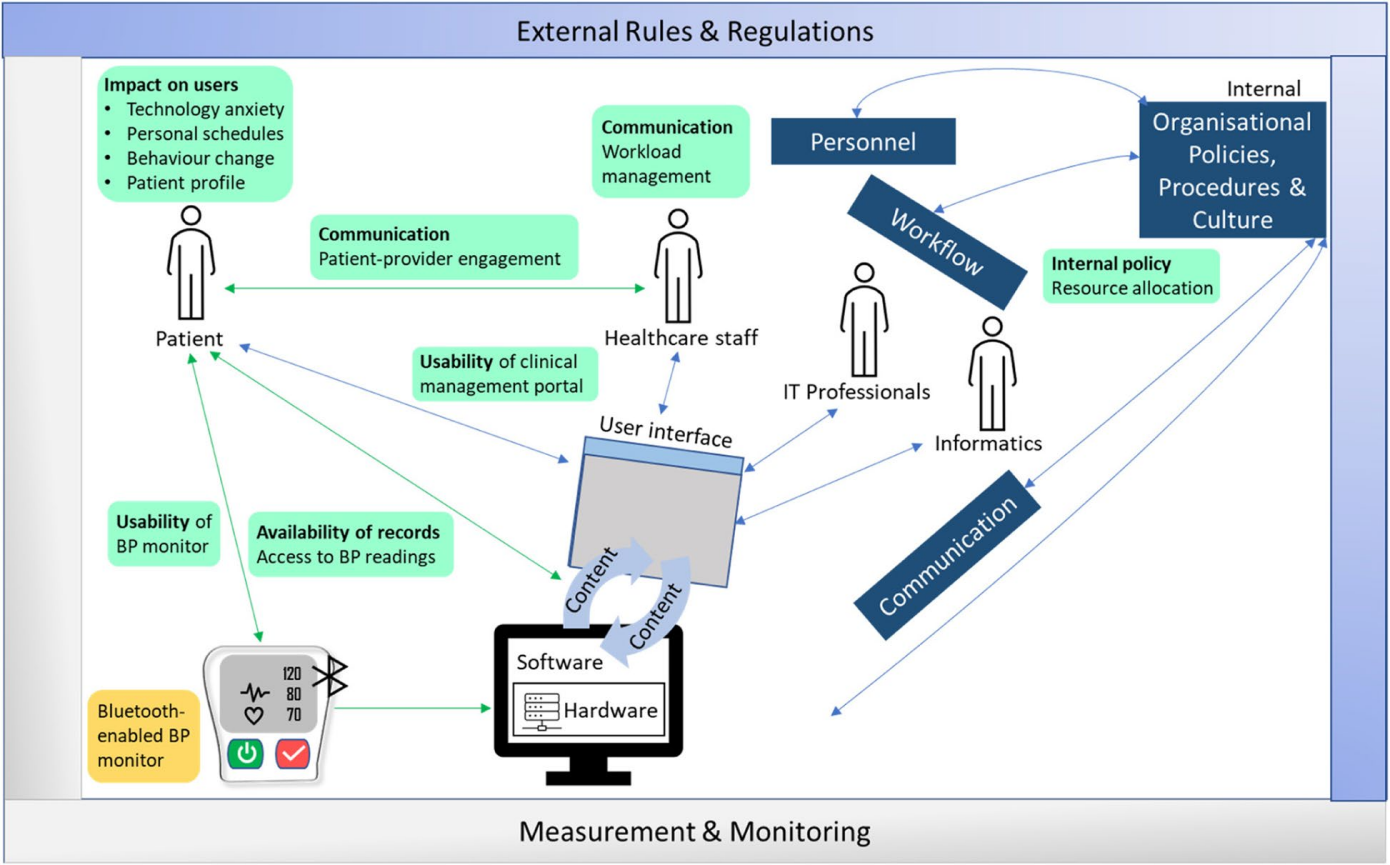
Study findings mapped to the socio-technical systems model. Green boxes represent emergent themes and subthemes, green arrows represent additional relationships identified from the study

We reported our key findings classified into themes presented by the applicable STS dimensions. The emergent codes, categories, and themes were represented in a coding tree chart (Fig. 2). Representative quotes are illustrated in Additional file 2 and Fig. 3 summarizes the study findings mapped to the STS model.

### Hardware and software

#### Usability and functionality

Six patients commented that the devices were fast and easy to use, with clear indications and instructions by the care team. Four patients were below 50 years old, two were above 60 years old, and all were in full-time or part-time employment.

Nevertheless, patients encountered technical challenges with the devices. Four patients were frustrated that the cuff did not fit them well. F035 (male, 67 years old) lamented that it was “really big, stiff!”, and F009 (female, 47 years old) was frustrated as “it keeps dropping down…By the time I can take the measurement, I think my blood pressure shoot up already.” S003 (care manager) acknowledged the issue, “So the first batch they were all quite stiff, very hard to apply. So no matter how you do it, it’s always quite loose…” Cuffs were subsequently replaced for some patients during the initial stage of the trial to prevent inaccurate readings and loss of motivation from patients.

Many patients also experienced challenges with the gateway, which they referred to as “phone”. They described the connection issues and that it would switch to flight mode unknowingly as the option was close to the power button, thereby affecting the transmission of readings. Some found the gateway cumbersome as it added to the number of devices they had to manage. The care coordinators, whom patients usually approached for troubleshooting, recalled that some patients withdrew from the trial due to the gateway issues. S001 escalated some issues to the vendor, while S007 was concerned that these performance issues might affect patients’ confidence in the intervention and relationship with the care team “like they lose trust in our treatment because the devices don’t work well or not as accurate as how they expect it to be.”

When asked about how the issues could be ameliorated, most patients, care managers and care coordinators expressed preference for a more user-friendly machine that could integrate BP measurement and data transmission more seamlessly. Patients who provided suggestions were all professionals with tertiary education, including IT professionals, managers, and teachers. F022 who was working in senior management, remarked that the intervention was outdated and advised the team to obtain user experience feedback as the intervention was being implemented and enhanced.

User experience is something that’s very important…put on a hat of a profile of the client, whether it’s a particular age group, then you try to empathize how that person feels…Maybe some of them are not so technologically-savvy…go through with them…and make it simpler. Interaction can be just a one-touch button, to make it more user-friendly. Things should be intuitive. (F022, male, 64 years old)

### Clinical content

#### Availability of records

The transmitted BP readings were only accessible through the web-based clinical management portal by the HCPs, and patients did not know how to retrieve the history from the BP monitor. Therefore, F096 and F099 kept their own record through methods such as taking a video or photo of the readings displayed on the monitor, or manually in a book.

Three patients expressed desire to have access to their readings, so that they could share with other healthcare providers, or monitor the BP trends and motivate themselves to adopt a healthier lifestyle.

I think it’ll be good if you can aggregate the readings over a month and say…I think you’re doing very well or not doing too well, and maybe nudge you, you may like to exercise or you might want to control your diet, less salt…I think it is a behavioral thing that can nudge you to a more healthy lifestyle…I think every participant would benefit. (F022, male, 64 years old)

They also suggested having an integrated mobile application where patients could readily communicate with the care team or access their BP data and other useful information, such as contact numbers of the care team and frequently asked questions. HCPs interviewed concurred as patients could feel empowered and a sense of ownership when they have access to their own data.

A concern arising from having data access, and with consultation and medication titration being done over the phone, was data confidentiality as unauthorized personnel may impersonate as HCPs. Patients proposed verification methods, such as two-factor authentication or using the device serial number, to ensure that the call is genuinely from the polyclinic.

### Human–computer interface

#### Usability of clinical management portal

The care coordinators and care managers who regularly used the portal for enrolling patients and monitoring patients’ BP noted that its functions and interface were manual and not user-friendly. This slowed their work down and they took some time to adapt to it, affecting the feasibility of the intervention.

Not the easiest to navigate… it’s not very neat and tidy. The buttons are like everywhere… So not the most intuitive to use, took me awhile to get used to it. (S003, female, care manager)

It’s not that bad but it’s not very straight-forward…But of course can improve much more… the speed is not very fast... Due to PDPA [Personal Data Protection Act], it keeps logging us out halfway… It feels like you wasted all your documents. A little bit complicated... A lot of info. (S007, female, care coordinator)

### People

#### Impact on users

The participants described the emotional to physical to behavioral impact that they experienced since they started using the intervention.

##### Caused technology anxiety

Three patients, who were above 60 years old and were retired or working part-time, described how they became more anxious since participating in the trial. Their concerns were—afraid to see high BP readings, errors with transmission of readings, unable to reach polyclinic hotline, and found it a chore to remember to measure BP. Two of these three patients had uncontrolled BP (≥ 140/90 mmHg) after 6 months of trial participation. Staff also recalled encountering anxious patients, some of whom even withdrew from the trial.

[It is] okay but also sometimes gives you anxiety. Like if high, you [get] scared… Sometimes it could be a chore, you have to remember to measure. (F099, female, 64 years old)

Sometimes the hotline can be very busy, and if you are very urgent, you can’t get through, you can get panicked. (F122, female, 73 years old)

Three patients also feared losing or damaging the BP monitor, as they perceived it to belong to the government and compensation would be a consequence. Due to this, F007 (male, 47 years old) kept the devices in the box after each measurement, which became a barrier and “makes it more cumbersome” to take regular BP readings since it was kept out of sight.

##### Personal schedules

Most patients and HCPs appreciated the convenience brought about by the intervention. It saved patients’ clinic visits, travelling and waiting time, especially for the working individuals. They could also monitor at a time that suited their schedules. Patients recounted that they no longer had to record BP readings manually on a monitoring sheet, and HCPs could monitor the readings from the portal easily without having to wait for the patients to bring the physical copy at their next clinic visit.

One thing is because I can easily monitor it at home, only one week once, out of my own convenience, how I want to check it. Sometimes I do it after I came back from work, after I have a wash, relax myself… You just have to press a button, it got transmitted... for you. Rather than you have to jot down…the timing… (F096, female, 46 years old)

During the intervention period, patients received scheduled and unscheduled teleconsultations from the clinic to follow up on their BP readings. While F029 (male, 49 years old) liked that the timing was flexible as “at that time [when they called] I was busy so I rescheduled…”, three other patients described how scheduled calls were not punctual despite having made appointments, or that the calls did not match their work schedules, resulting in rejected or missed calls.

Office hours and sometimes, you are in the midst of doing something, and then the call come in. So, it’s like a lot of interruption…Your care team, they have a job to do. They follow up and all. I mean that’s good. But for people working, I think it’s a little bit inconvenient. Especially the few times when they called me, I was in the meeting with my boss. So, I have to say, “No, I’m busy now. I cannot talk to you." (F119, male, 58 years old)

The care managers, who usually made the calls, mentioned that calls to most patients were completed smoothly. However, there were instances of unfriendly remarks from patients or unresponsiveness and they had to make multiple calls to patients which added to their workload.

Sometimes they work same times as us…they will need to keep the hand phones [aside] so they can’t answer… It’s not that they don’t want to [answer]. (S003, female, care manager)

##### Behavior change

Six patients, mostly working individuals, reported that the intervention had helped instill a habit, routine and discipline of measuring BP regularly, which would otherwise not be possible even if they had owned a BP monitor.

Even though you can buy [a BP monitor], you don't have the discipline to check, but with this thing, you know that you have to check one week once, so there is no excuse that you don't have to check…that is one thing good about it... The responsibility is there... (F096, female, 46 years old)

Patients became more motivated and improved their self-management and health literacy of hypertension from the advice provided by the care team. They grew more aware of their own triggers of BP fluctuations and ways to maintain a healthy BP in terms of diet, exercise and emotions. F022 felt that participation in the trial allowed patients to have “more ownership of managing their own healthcare”. S003 noted that patients had shown “good outcomes”, and S007 found that whether patients were new to home BP monitoring or not, the intervention “gives them an exposure to start monitoring themselves…Empowered, maybe.”

##### Patient profile

The participants identified the patient profiles that are more suitable for the intervention than others. These include personal schedules, adaptability, age and digital literacy, health literacy and social support, and physical condition. They felt that the intervention would be more appropriate for certain individuals – busy, retired, adaptable or accepting of technology, possess some level of health and digital literacy regardless of age, or have social support if they are unable to self-manage. Participants also highlighted that the intervention would benefit those with poor BP control and had a risk or history of stroke to keep them motivated and prevent their condition from worsening.

Among the study participants, those who were younger and received tertiary education were more accepting of teleconsultation and phone medication titration. Contrastingly, the older patients preferred traditional face-to-face mode of care for better communication or when discussions involved medications. F035 preferred physical consultations as he could obtain the test reports from the clinic and that “through the phone, maybe communication breakdown” as the elderly may have hearing difficulties. HCPs agreed that physical consultations might be more useful for elderly patients, or for those who required monitoring of medication compliance. S003 felt that “patients may feel more confident to have a face-to-face [consultation] especially those like elderly patients” and that people in Singapore are generally “not used to talk to a person over the phone asking many health-related questions”.

### Workflow and communication

#### Communication process

The participants discussed several aspects of the intervention relating to internal workflows and engagement between patients and HCPs.

##### Workload management

Through the intervention, HCPs could receive patients’ BP readings remotely and conduct phone medication titration quickly if warranted. This raised conflicting views from HCPs about the impact on their workload.

In the initial stages of the trial, S005 (female, care manager) had to “find pockets of time” to check each patient’s BP readings monthly for fluctuations through the portal and phone calls, “whether it is urgent or not”. Subsequently, a Jobstack function was created and helped the healthcare team prioritize certain patients for closer monitoring and unscheduled teleconsultations. This freed up some time for them to focus on other tasks. Since then, S005, S006 and S007 saw better workload management as they could spend their clinic time attending to patients with more complex needs, while conducting phone medication titration and teleconsultation for patients with stable BP.

At individual level, the time that I usually spend for my patient [usual consult] compared to now for this [intervention], it has reduced a little. But overall, if I see it as a whole, for the 120 patients, it does help to ease out the appointment for other patients. If I get a lot of them to be on teleconsult, so I free up some of the appointments for other more complex cases. (S006, male, family physician)

On the contrary, the intervention become overwhelming for some HCPs. S002 found an increase in workload as she had to manage patients with clinic appointments and phone follow-up with the trial participants concurrently.

It’s quite packed every day… Sometimes [the time] I spend [on] the call is very long, so the patient actually in the clinic was waiting. These are the difficulties. (S002, female, care manager)

One family physician felt that the effort involved in teleconsultation and phone medication titration in this pilot trial was not acknowledged but was hopeful that the workload might improve in future.

All [done] remotely, [patients] don’t have to come back. So in a way, [they] save time, right? Because they don’t come in and take our slot. But the bad thing is that it is not accounted for, it’s just extra work... Maybe in the initial phase, there will be increased workload, but in the long run once everyone is optimized… there may be a decrease in the workload. (S004, female, family physician)

##### Patient-provider engagement

The patients and HCPs reflected on the engagement between each other relating to the relationship built and support provided during the intervention.

Patients reported a sense of security, as they knew that they were being monitored closely by the healthcare team, who would alert them of any irregular or missing BP readings. F035 (male, 67 years old) even exclaimed “I feel happy!” when he received an unscheduled teleconsultation for a rise in BP. This also motivated him to measure BP regularly and look out for triggers. HCPs concurred that issues with patients’ BP could be addressed in a timelier manner through this intervention as the healthcare team could intervene quickly, if needed, through a teleconsultation or phone medication titration. HCPs felt that their relationship with patients improved, as the ready technical support rendered by the care coordinators, frequent phone calls and encouragement by the doctors and care managers, and BP improvements contributed to the building of rapport and between patient and HCPs.

Conversely, four patients were expecting to receive feedback on their BP readings from the healthcare team during the trial, but they did not seem to have received any phone calls. Two were aged 47–49 years old and two were aged 64–73 years old. This affected their confidence in the patient-provider engagement, as F007 wondered “if someone is looking at my records or just for the sake of collecting data”. They preferred more regular interaction with the healthcare team to understand HCP workflows in relation to monitoring BP readings, provide feedback on the process and receive advice on their BP readings and lifestyle management.

F029 (male, 49 years old) recounted that she had to return to the clinic to refill her medications as the doctor increased the dosage over phone medication titration. However, the pharmacy was not informed and she “had to wait for quite some time because I guess they need to clarify with the doctor… So if that link is there, it’s more convenient.” This also highlighted that the healthcare teams need to work with other clinic staff who might not be part of but still support the core team, such as the pharmacist.

### Organizational policies and culture

#### Internal policy

HCPs interviewed, especially the care managers and care coordinators, were concerned about the limited manpower and time available, which would also affect the workload of the intervention. They wondered whether adjustments to internal allocation of resources would be possible in order for the intervention to sustain.

If we have a mobile phone to call the patients, if patients keep on texting or messaging, then the nurse will be very stressed also. So I don’t know whether in the future [there] will be a coordinator [who] have some nursing background [and] able to man the phone… (S002, female, care manager)

Maybe give us a bit of extra time every day to just handle things like this. I would say, minimum maybe 15 minutes a day? If not, half an hour would be better, to go through the cases. (S004, female, family physician)

During the initial recruitment stage, like on standby, we can have more care coordinators and care managers in the same area to prepare to recruit patients. Because sometimes when it’s just one care coordinator, it gets piled up more and more. Then every recruitment could get very long, half an hour to an hour... But at the same time, it’s quite hard because the doctors and care managers also have to see other patients… So that part in workflow, it’s more on team communication also. (S007, female, care coordinator)

## Discussion

The purpose of this study was to assess the barriers and facilitators to the implementation and use of the PTEC-HT BP telemonitoring system with teleconsultation, to inform future expansion of the intervention. Our analysis showed that the technical, human, workflow and organizational factors involved were complex and interdependent, and the STS model provided a comprehensive framework to understand the relationship between these dimensions.

Our study findings further substantiated the patient satisfaction survey results of the PTEC-HT pilot trial, in which patients agreed that it was convenient to record and share BP measurements with the healthcare team, were motivated to monitor BP weekly and were satisfied with the advice provided through teleconsultations [23]. Overall, patients had a positive experience using the intervention although they encountered technical difficulties and expressed concerns relating to data security and technology anxiety. Through implementing the intervention, HCPs found that it helped with workload management and useful for patients, despite challenges with usability and resource allocation. Suggestions to overcome these obstacles were provided, for the intervention to be feasible and sustainable in Singapore’s primary care setting.

### Comparison with prior work

The themes that emerged from the current study were largely consistent with the literature which reported that technological usability, reduction of in-office visits, positive impact on self-management and improved patient-provider relationship were the most frequently reported facilitators for adoption of telemedicine [30]. However, several differences exist.

#### Hardware and software, human–computer interface, people

Our study participants found the intervention easy-to-use, and patients could save time travelling to and waiting in the hectic polyclinic setting, or recording BP readings manually. HCPs also had quicker access to BP readings and could conduct teleconsultation, if necessary, thereby improving their efficiency. Fletcher et al. reported that patients liked the ability to be able to monitor BP whenever they wanted as they were more relaxed outside the clinic environment [31]. This was reflected by a patient in this study who attributed the convenience to having the ability to monitor any time she preferred in a week.

Patients in our study saw changes in their attitudes and behaviors in managing hypertension, like previous studies conducted in the US and UK [16–19, 32, 33]. They appreciated that the intervention helped to form a habit of regular BP self-monitoring and improved their self-management. Improvements in BP readings and lifestyle behaviors coupled with timely feedback from the care team, increased their awareness, motivation and sense of responsibility for their own condition.

Participants experienced technical challenges with using the equipment and clinical management portal. Firstly, issues with the cuff and gateway and having to handle multiple cumbersome devices caused frustration and affected patients’ motivation to continue the intervention. They preferred the functions of measuring, recording and transmission of BP readings to be integrated in one machine. Similar to interventions targeting home blood glucose and BP telemonitoring for patients with diabetes, the equipment was not “plug and play” for some patients and higher digital literacy and frequent assistance from the care team was needed to overcome these obstacles [32]. Secondly, while HCPs were able to receive patients’ BP readings quickly through the management portal, they did not find it intuitive. Additionally, they had to check through BP readings of all patients in the initial stage. These translated to additional workload and time to navigate the portal and affected their efficiency, until the Jobstack function was created to help with prioritizing patients for closer management.

Patients and clinicians were found to be uncomfortable with the interpretation of self-monitoring measurements due to the difference between home and office readings [31]. Some of our study participants were ambivalent due to the technical challenges, while some perceived home BP readings to be more accurate than office readings which could otherwise be confounded by white coat hypertension. Besides, the Ministry of Health Clinical Practice Guidelines for hypertension indicated home BP monitoring for monitoring BP in diagnosed patients. The guidelines proposed that home BP monitoring is cheaper, more widely available, easily repeatable and shows day-to-day variability, and it can be offered to committed patients to improve treatment adherence via positive data feedback [34].

#### Clinical content, people

Patients in the current study wished to have access to their data for sharing with other HCPs or self-management, and some form of feedback or advice even if their readings were satisfactory. These could encourage them to become more engaged as active managers of their healthcare thereby facilitating self-management.

However, while most patients were comfortable with data being shared between patients and HCPs, some were concerned about data privacy and security especially when the intervention involved medication titration. This is a common concern reported in the literature, and it was expected due to the health data security breaches previously reported in Singapore [22, 35, 36]. Singapore has the most comprehensive telemedicine guidelines in Southeast Asia that provides advice on various aspects of telemedicine usage, and is comparable with other countries [37]. An example is the Personal Data Protection Act (PDPA) mentioned by S007, which governs the collection, use, disclosure and care of personal data in Singapore. Unfortunately, it created a barrier for HCPs as they were logged out from the portal frequently possibly to prevent unauthorized access. These external regulations can help to inform the development of organizational policies, which would be important for allocating budget and stipulating policies and workflows for authentication and data backups, and help HCPs provide safe and effective care to patients.

#### People

There was variability in the use of the intervention by certain populations in this study. Older patients experienced technology anxiety as they were afraid to see abnormal readings or were worried that the readings did not get transmitted. They also preferred face-to-face consultations possibly for the human connection [38, 39]. Contrastingly, younger patients or those with higher education level and busy lifestyle welcomed telemonitoring, teleconsultation and phone medication titration. Perhaps a common issue in Asia, a Malaysian study also found that older patients found the telemonitoring device unfamiliar and had difficulties using it [40]. This could be due to the different levels of health literacy, digital literacy and technology acceptance, as shown in a study on perception towards digital health among elderly Singaporeans [41]. Sin et al. found that only a slim majority of patients with diabetes and/or hypertension were willing to adopt telemedicine, and care satisfaction with using telemedicine was a significant factor [35]. Although Singapore is technologically advanced, levels of comfort with health technologies still vary considerably.

Participants also proposed higher utility of the intervention by specific populations, and this suggests the need for patient segmentation to craft and target strategies to assist patients with various needs to achieve maximum benefit. The personae proposed by Low et al. or the Bloem-Stalpers model could be considered to segment patients according to their socioeconomic and sociodemographic characteristics, acceptance of technology, or acceptance and perceived control of their condition [39, 42].

#### Workflow and communication, people, organizational policies and culture

Whereas patients and HCPs were reported to be concerned about telemedicine hindering the patient-provider relationship [30], those in our study felt that the frequent support and encouragement provided to the patients improved this relationship. The intervention complemented the relationship, rather than interposing as an intruder, by providing timely and accurate information via telephone calls in between clinic consultations [43]. Patients felt reassured that their health was being closely monitored, through the quick feedback from the healthcare team especially when abnormal readings were recorded. However, the practice of conducting teleconsultations or tele-support was not consistent as although patients were informed upon enrolment to expect calls from the healthcare team during the intervention period, some did not receive any and might perceive this as a lack of accountability by the institution.

While other studies mainly focused on the doctor as the provider of the intervention, team-based care was provided at the polyclinic as teamlets. However, one patient experienced confusion as there was a lack of communication with the pharmacists, who usually supported the teamlets. Coordinating workflows with other support staff ensures patient safety in the form of timely supply of patients’ medications and can also help to maintain patients’ motivation for the intervention.

Although there was divergence of HCPs’ opinions on whether the intervention helped with workload management, they agreed on the need to allocate more resources to prevent burnout, and for the intervention to be sustainable in the long run. Although geographical barrier is less of a concern in Singapore than in other countries where healthcare may not readily accessible, the COVID-19 pandemic has created an invisible barrier between HCPs and patients in the form of safe distancing and reallocation of clinic manpower to support COVID-19 operations and other priorities. Telemedicine, with adequate planning of resources and workflows, can bridge this barrier by sustaining BP management and providing care to patients even in times when physical visits are not possible [44].

The study populations of previous studies were mainly from the West and may not be translated to the Singapore or Asian context. The interventions in these studies included monitoring, medication titration and contact initiated by patients. However, the PTEC-HT intervention required HCPs to initiate contact with patients through scheduled or unscheduled teleconsultation, which was not available in the referenced studies. This provided sufficient support right from the start, especially for patients with technology anxiety or low health or digital literacy. Teleconsultation can provide reassurance, improve patient education and health communication and contribute to behavior change, addressing the areas for future research raised by Fletcher et al. [31].

As we focus on patient-centredness in the implementation and use of telemedicine interventions, we can incorporate principles of service design and human factor approaches to achieve greater impact and better experience for our patients and HCPs [45, 46]. Study participants suggested that it is imperative to obtain and incorporate user feedback earlier in the development of user-friendly telemedicine interventions which prioritize effectiveness, patient safety, data confidentiality and the needs of patients and HCPs.

### Strengths and limitations

This study was conducted within a quasi-experimental trial using qualitative methodologies. It provided insights into the views of patients and HCPs on the implementation and use of BP telemonitoring system with teleconsultation in a real-world setting. Integration with routine clinical practice and the ability to adapt features of the intervention encouraged uptake by both patients and HCPs. Using semi-structured interviews allowed us to build rapport with the participants and obtain rich data about their experiences. We interviewed patients and HCPs to obtain a diversity of views as they played different roles in the process.

Applying the STS model in the analysis allowed us to study the intervention on a more holistic level and identified some complexities and important considerations as we plan and improve the implementation and use of telemedicine interventions in primary care. The dimensions of “external rules, regulations and pressures” and “system measurement and monitoring” were not addressed in this study as participants did not express any related views. Future research could consider interviews with developers of the intervention and healthcare administrators to understand the impact of any external forces and the expected outcomes of the intervention.

There have been several other models developed to study health technologies [27, 43], but few provide a holistic framework to do so. The exception is the nonadoption, abandonment, scale-up, spread and sustainability (NASSS) framework by Greenhalgh et al., which is very flexible and useful to study technology implementation at the micro, meso and macro-level [47]. While the current study was more limited in scope which focused more on the users’ perspectives and the impact of the intervention on the users, communication and practice workflow in one polyclinic, a longitudinal qualitative approach using the NASSS framework could be used to study the adaptation, scale-up and adoption or non-adoption of the intervention over time in more polyclinics. The scaling of the PTEC-HT program with more integrated features is currently underway [48].

Notwithstanding the efforts to obtain a representative sample of patients to reflect demographics of the clinic, ethnic diversity was limited. Self-selection might also have occurred from voluntary participation. Compared to other participants in the PTEC-HT pilot trial, patients in this study were younger (mean age 50.5 vs 56.3 years) and a larger proportion attained tertiary education qualification (53.8% vs 25.2%), and they might have been more willing and able to share their views.

The patient interviews were conducted when the patients had completed the 6-month trial in hope to maximize their experience and elucidate their perspectives of what did or did not work. However, there was a possibility that some patients could not recall details from the early months of their participation accurately. Future research may also involve views from patients who rejected participation or withdrew from the trial to understand their reasons and concerns. As the HCPs were part of the teamlet tasked to implement the intervention, they might have been supporters of the intervention so their views might not be representative of other primary care HCPs.

As this was a qualitative study conducted in one public primary care clinic in Singapore before the COVID-19 pandemic, transferability of findings to other primary care clinics or non-primary care settings is limited as operational procedures may differ between institutions. However, the challenges identified from this study, such as digital literacy and data security, are still largely relevant even as we are adapting our healthcare services since the pandemic happened [49].

## Conclusions

Our study provides comprehensive perspectives on the barriers and facilitators to the implementation and use of a BP telemedicine intervention in a complex fast-paced primary care setting. It can engender various benefits and challenges to patients, healthcare professionals and the healthcare system. An intervention that is well-designed and successfully implemented can be an enabler to improve healthcare delivery, patient journey and safety, as well as avoid unplanned hospital admissions and visits. It is hoped that the insights gleaned can help to guide various stakeholders understand the importance of the various dimensions as they embark on telemedicine program design and implementation.

## Supplementary Information

**Additional file 1.** 

**Additional file 2.** 

## Data Availability

The data generated and/or analyzed are available upon reasonable request. The data request can be sent to Clinical Research Unit, National Healthcare Group Polyclinics, Singapore; Email address: NHGP_CRU@nhgp.com.sg.

## Abbreviations

BP: Blood pressure
COREQ: Consolidated criteria for Reporting Qualitative research
HCP: Healthcare professional
PTEC-HT: Primary Technology-Enhanced Care Hypertension
STS: Socio-technical systems
