# Supplementary material for: Implementation and use of technology-enabled blood pressure monitoring and teleconsultation in Singapore’s primary care: a qualitative evaluation using the socio-technical systems approach
Source: BMC Prim Care. 2023 Mar 16;24:71. doi: 10.1186/s12875-023-02014-8 (PMC10018584; doi:10.1186/s12875-023-02014-8)
Supplement: Supplementary file 1 — Additional file 1. [file 12875_2023_2014_MOESM1_ESM.docx]

**Additional file 1: Patient and healthcare professional topic guide**

Table S1: Patients

| **Opening Questions**   - Can you tell me what it is like for you to have high blood pressure? - Can you tell me why you signed up for this study? - Can you tell me about your experience of your chronic disease management with your care team? |
| --- |
| **Experience of participating in the study**   - Can you share about what you liked about the study? - Can you tell me about anything you did not like? - Were you monitoring your blood pressure before entering this study? - Can you tell me about your experience of monitoring your blood pressure using telemonitoring equipment? How is this different from what you have been doing before entering this study? - Can you share about your experience with using the gateway device?   - Time taken to start the device   - Usability - Have you used the BP machine outside of home? Can you tell me your experience?   - Have you used it overseas? - What do you feel are some of the advantages of telemonitoring of blood pressure? - What do you feel are some of the disadvantages? - How do you feel about your blood pressure now? - Has participating in this study made you more aware of monitoring your blood pressure? |
| If this programme becomes available permanently, would you choose to continue using the telemonitoring equipment and self-monitor your blood pressure?   - Why - Why not? |
| If this programme becomes available permanently, would you choose to continue using the tele treatment (consultation and medication adjustment) and self-monitor your blood pressure?   - Why - Why not? |
| Would you recommend this telehealth programme to other people with high blood pressure?  Do you have any suggestions for improvement? Please elaborate. |
| **Self-Monitoring Experience**   - Can you tell me what it was like to learn to use the BP machine at home? - Can you share what it has been like to monitor your blood pressure yourself? - How did it make you feel? - Can you tell me about any concerns that you might have about monitoring your blood pressure? - Were there times that you wanted to drop out from this programme? - What will motivate you to continue monitoring your blood pressure? |
| **Adherence to monitoring schedule**  (You were asked to monitor/record your BP at least once a week over the study period of 6 months)   - What has it been like for you to try and monitor your BP at least once every week? - How did you feel about the instructions given regarding this? - Were there any difficulties in following the instructions and monitoring BP? - Can you tell me about any time you had to skip your BP monitoring? |
| **Teleconsultation Experience**   - Can you tell me if the teleconsultation appointments were convenient? - How easy was it for you get an appointment? - How did it make you feel? - Can you tell me about any concerns that you might have about teleconsultation? - Did you have any changes to your prescribed medications during these 6 months?   - How was it done?   - How was this different from your previous experience with changes to your medications?   - How did you feel about having your medicines adjusted through the teleconsultation? - I am interested in hearing about your experience of getting medicine adjustments over a phone call, can you tell me about it? - Besides scheduled calls, have you received any other calls from the clinic? |
| **Feedback Messages/Phone call by care managers**   - What did you think about the feedback you received on your readings during your teleconsultations? |
| **Other healthy changes**   - Can you tell me about anything else that you tried to change in your lifestyle to manage your BP? |
| **Additional points**   - Are there any other things that you feel can enhance your experience, for example a chatbot? - You were given a number to call in case you needed technical support. Did you make use of that? - How do you feel about the support provided during the study? - Do you feel that it will be helpful if you could also clarify your medical doubts by calling this contact number? |
| **Closing**  - Is there anything else that you will like to add that is not already covered?  - Thank you so much for your time and participation. |

Table S2: Healthcare professionals

| **Experience of participating in the study**   - I am interested in hearing about your experience of providing care to the participants of this study, can you tell me all about it?   - Can you tell me about your role in this study? - How is your experience different from usual care? Can you share what you liked about the study? - Can you share what you did not like about the study? - What would be some of the advantages of telehealth (BP monitoring, teleconsultation, medication adjustment) in the management of blood pressure? - Can you share some of the disadvantages? - How has your experience been with patients under this study?   - Could you elaborate about how this study affected relationship between patient and the healthcare provider? - Did you have any interesting experiences arising from the study? |
| --- |
| Do you think patients would continue using telehealth to manage their blood pressure if it was still available after this study? Why or why not? |
| Would you recommend telehealth to manage blood pressure to your patients with high blood pressure? |
| Do you have any suggestions for improvement? For e.g. when and under what situations would a chatbot be useful? Please elaborate. |
| **Onboarding (For Care Coordinators)**   - How was your experience in enrolling patients for this study? - How did you find the clinic management portal? For e.g. navigation, speed, format, connectivity. - How was your experience in teaching patients about using the BP machine? - What were some of the difficulties that patients faced? - What were some of the difficulties that you faced? |
| **Teleconsultation Experience (For Care Managers)**   - How was your experience of teleconsultation with your patients? - Can you share what you liked about teleconsultation sessions? - Can you share what you did not like about teleconsultation sessions? - What would be some of the advantages of teleconsultation? - Can you share some of the disadvantages? - How was it to interact with patients via this platform as compared to usual clinic visits? |
| **Medication Titration Experience (For Doctors, Care Managers)**   - How was your experience of medication titration in this study? - Can you share what you liked about medication titration in this study? - Can you share what you did not like about medication titration in this study? - What would be some of the advantages of this way of titrating medication for hypertensive patients? - Can you share some of the disadvantages? |
| - Would you recommend your friends or family to enrol in a similar programme? Why or why not?  - What do you think is the value of this programme to patients? |
| **Closing**  - Is there anything else that you will like to add that is not already covered?  - Thank you so much for your time and participation. |
