# Supplementary material for: Implementation and use of technology-enabled blood pressure monitoring and teleconsultation in Singapore’s primary care: a qualitative evaluation using the socio-technical systems approach
Source: BMC Prim Care. 2023 Mar 16;24:71. doi: 10.1186/s12875-023-02014-8 (PMC10018584; doi:10.1186/s12875-023-02014-8)
Supplement: Supplementary file 2 — Additional file 2. [file 12875_2023_2014_MOESM2_ESM.docx]

Table S3: Characteristics of participants

| **Participant ID** | **Gender** | **Age / Profession** |
| --- | --- | --- |
| F007 | Male | 47 |
| F009 | Female | 47 |
| F021 | Male | 58 |
| F022 | Male | 64 |
| F026 | Male | 49 |
| F029 | Male | 49 |
| F035 | Male | 67 |
| F096 | Female | 46 |
| F099 | Female | 64 |
| F110 | Male | 35 |
| F118 | Female | 67 |
| F119 | Male | 58 |
| F122 | Female | 73 |
| S001 | Female | Care coordinator |
| S002 | Female | Care manager |
| S003 | Female | Care manager |
| S004 | Female | Family physician |
| S005 | Female | Care manager |
| S006 | Male | Family physician |
| S007 | Female | Care coordinator |
| S008 | Female | Nurse clinician |

Table S4

**Dimension: Hardware and software / Theme: Usability and functionality / Category: Challenges with device, suggestions for device**

| **Code** | **Challenges** | **Suggestions** |
| --- | --- | --- |
| Gateway issues | Already change it, so far 3 times already. Yeah it costs [many] batteries… If normal like the Eveready [brand], the battery cannot last one week. Yeah, so I changed to Energizer. (F009)  Initially like battery weak, there was an error message, but there’s no reference guide, to say this error code means what…(F021) | If you want to roll out for mass…and linked to your smartphone so the upload can be faster and also integrate apps like your tracker or your smartwatch, that can give you more inputs into the data… (F022) |
| Gateway issues | The airplane mode shouldn’t be there! The upper part is off the machine, then airplane mode… There may be some old people… you can press wrongly… Then keep on ringing, don’t know what to do. Can get frustrated… (F035)  I would say, technologically outdated. I mean now you have your own smartphone. You can very easily put an app [application] using Bluetooth, and I think the thing is a bit slow in transmitting, so usually there’s a long wait... (F022) | More user-friendly gateway, or rather, maybe just incorporate into the BP set so that the patients don’t find it that more difficult or what. They can just put at the bedside, compare to current one. (S005)  Maybe integrated with the watch? Because watch will be wearing it every time…can measure the BP at the same time. So keep on uploading without our intervention to keep on syncing right… the best is to integrate everything inside one machine. (F007) |
| Cumbersome | I don’t find any value-add because why? If I go out, I have to carry 2 machines. One phone, one machine, and my own phone. End up, I got a lot of gadgets… I have many process[es] to do. So I think it’s not very productive. (F119)  They don’t understand why they need the gateway first before connecting to the BP [monitor]…So, technical part that could be sort of a bit of a downfall in convincing patient to continue...They find it troublesome to monitor, troublesome to use the machines…they just return it to us because mainly of the gateway issues. (S007) | Like it’s just one machine, there is some satellite in it that can just direct link up to the system. Because so many meter-devices, like patients also get confused. Then charging-wise also, every device has batteries…Because sometimes during the measurement, it takes very long for it to connect to the gateway, for it to start up…it would be good if they can be like a smartphone…Faster response, less device issues, and more direct, without another gateway to connect. (S007) |

Table S5

**Dimension: Clinical content / Theme: Avaialbility of records / Category: Access to BP readings**

| **Code** | **Challenges** | **Suggestions** |
| --- | --- | --- |
| Transmitted readings not accessible by patients  Alternative ways of recording readings | When you measured, you don’t know [whether] the data [was] successfully transferred… cannot go back to look as it doesn’t have the time and date… (F026).  Because sometimes, you already transmitted [the readings] then it’s cleared... Record down better. I can refer, I can see clearly…(F035)  I take a video, after that I take a photo, every reading that I took. (F096)  I have a book, I will record…. Otherwise in the machine…if I don’t record, I cannot see the actual reading. (F099) | **Sharing of data**  The best is that…whatever is recorded in the gateway they can send to me through an email, then don’t need to keep a record… In future if I go to any hospital, I can tell them I got this record. (F009)  So if possible maybe share the data if you can…or some PDF file or just picture…sometimes you need to give the doctor all these…then [the doctor] doesn’t need to say, “I don’t have your data, I need to check my computer.” (F026)  I think it’ll be good if you can aggregate the readings over a month and say “I think you’re doing very well or you’re not doing too well”…nudge you to say “maybe you may like to exercise, or you might want to control your diet, less salt”…I think these nudges are a behavioral thing that can nudge you to a more healthy lifestyle which I think not just me, I think every participant would benefit... (F022) |
|  |  | **Mobile application**  Apps is the best. That’s where I can get the source of all the contact numbers. Even my BP information… Or even if the care coordinator has some issue, you can just prompt a message… push notifications. (F007)  Maybe can see our blood pressure tracking… we can use the app to do everything… maybe some app they already have the question-answer... So maybe from there we can get the answer [to questions] and then we no need to call. (F009)  If you can have a platform that integrates all these things together, that would be good… more data points for the person and your researchers to look at managing health remotely… (F022)  If the app allows them to put in some comments, then that will actually be very helpful…Patients don’t have to come back physically. It’s a convenience for them, also easier for me…The patients don’t want to read out to me all their BP readings, everything’s all there for me to see already. (S003) |

Table S6

**Dimension: People / Theme: Impact on users**

| **Category** | **Patient quotes** | **Healthcare professional quotes** |  |
| --- | --- | --- | --- |
| Technological anxiety | I have [blood] pressure [readings], don’t know why the readings are not transmitted. I have to take out some time later to measure again. Because of this, I withdrew… I get anxious very easily, then blood pressure will rise again… (F118)  Sometimes the hotline can be very busy, and if you are very urgent, you can’t get through, you can get panic. (F122)  You have this phobia that, will I lose [the devices] inside the bus or not? It’s not your own property, it belongs to the government. So in a way, it adds to the kind of pressure to you, that you have to make sure you look after it carefully… (F119) | They feel that the readings for this project is… higher [compared to their own machine]. Yes, then they verbalised to me that they [felt] a little bit of anxiety. (S001)  We saw a few cases [who] are very jumpy patients… Monitoring the home reading to them it’s like a chore and makes them very stressful. So they withdraw from the study. (S002) | |
| Personal schedules | I don’t have to come down to the clinic, I don’t have to apply leave because…my leave is not many so… it’s good, it’s very convenient. Any time that it’s convenient for me to take [BP reading], I just take it. (F029)  The appointment [was] fixed at a certain time. So I waited… I think [they] only called half an hour later. Normally, I don’t carry the phone when I go for lessons…If the person calls around plus minus 5 [minutes], it’s ok... But half an hour later, it’s too much. (F021) | Some they don’t pick up call, then some feel very irritated… if let’s say you cannot contact the patient, you one day call 3 times, the next day call another 3 times – actually time [is] wasted. (S002) | |
| Behaviour change | It’s good. They give you the advice, then they let you know actually these 3 months your [average] blood pressure is at what range… So now must try to keep it up, as a reminder of myself – try to keep it up more exercise, and eat more healthily… (F009)  Join this programme, double-check and monitor my blood pressure… Sometimes higher, you can also look out [for] the reason. Is it [because] I eat salty [food] or [insufficient] sleep? Or maybe angry...So you need to control, to calm down… (F026) | I guess [the intervention] also helps them to understand better how home monitoring actually helps to benefit the control of their condition… it’s a little bit easier to convince them that your pressure is not well-controlled and need to increase medicine... they probably can also adjust things like their lifestyle… (S004)  They are more aware of their blood pressure… So sometimes I just tell them “Oh don’t need [to take more medicine], because doctor says no need, but you need to be more careful with your diet...” A handful of patients will say “okay I try to swim more, jog more [and] see whether I can bring down the blood pressure.” (S005) | |

Table S7

**Dimension: People / Theme: Impact on users / Category: Patient profile**

| **Code** | **Patient quotes** | **Healthcare professional quotes** |  |
| --- | --- | --- | --- |
| Occupation |  | Some people who can’t come back to clinic so often, like those young working adults, with family...So that can benefit them…we telephone them instead, check if they’re good, then they just collect the medicine and just go off. So they only see the doctor once a year. (S003)  But really benefits those who has very busy schedule. So they just need that once a week recording and we can just monitor back end. (S007)  Or those who are always overseas assignment, that would be also a bit difficult. (S005) | |
|  | I am a retiree so I got plenty of time. I can adjust my time. For those who are working, maybe a bit difficult. (F122) | And maybe the retirees also, because they are very free right? So they can always monitor freely…we can always see their readings. (S007) | |
| Attitude towards technology |  | For patients who are…unable to actually adapt that quickly, then most probably it will be quite a hassle for them. (S001)  Their mindset must be there. If they are very resistant to medicine changes or they don’t believe in medicine, then telephonic consult won’t make a difference. (S005) | |
| Digital literacy | All those tech-IT savvy, I think that kind of young generation should be no problem. Older ones, I think would be difficult for them. But…if they are very eager to learn all these, I am sure they would be able to pick it up. (F007)  For elderly…maybe it’s quite difficult for them. Because they are not used to all this technology especially the hand phone, the gateway…all in English version and it’s quite hard for them…or rather they have a caregiver to assist them. If not… they will find it very troublesome. (F009)  Maybe for the older people, they might need their relative to help, so might be a problem for them... (F110)  For example my friend, he has higher knowledge, knows how to read, those English, I will recommend to him, it’s not bad. (F118) | Some patients they are elderly folks, but they are quite tech-savvy. So they are okay with the idea. (S001)  Must be some sort of IT-savvy, know how to operate the smart phone…even the BP set…patients [who are] not so receptive towards technology is harder… They also must [have] a bit of health literacy there. If not they won’t understand why you must change medicine over the phone… and know when to take action. (S005)  Only those who [have] higher level of health care literacy perhaps, higher level of empowerment, where they really are ready or they are already in action state of prioritising their health… then I would offer it to them. (S006)  Like the very frail, or not that well educated…if [they] don’t have good social support, and they just do everything on their own, sometimes it’s a bit dangerous to do things over the phone. We still want them to come back to see them. (S004)  We tend to say older [patients] will be more troublesome but may not be true. [There are also] some younger ones that cannot hear, cannot understand, very low literacy…If you involve a caregiver, somehow you’re not as effective as doing it on your own… The caregiver has to really have the time for you, has the love and concern for you... (S008) | |
| Medical history |  | Hearing must be there…So that can, get the message across, if not I say five milligram you think is fifteen milligram then will be “Oh, I say FIFTEEN, ONE FIVE” and then you hear as fifty, it will be a very disastrous consequence. So the hearing is very important. (S005) | |
|  | Patients who have high risk of stroke or have gotten stroke before… If they have [high BP], then all the more they have to monitor so that they can ask for advice or polyclinic can call them to adjust the medication so that the treatment or maybe the preventive measure can come a bit earlier. (F110)  Like those patients who just developed high blood pressure…maybe no. I think for people like with regular high blood pressure, will be very beneficial, because [the intervention will] make them more alert about the conditions, then they will faithfully take the medication…as indicated by the doctor... (F122) | Patients who have extra need to optimize their blood pressure…either they refuse to adjust their medicine because they refuse to believe their pressure is not well-controlled, or they just need tighter control to bring down the pressure. But for those very stable patients, probably not very suitable. Because we are going to be wasting a lot of time for the false alarms in-between. (S004)  [For a patient whose blood pressure is not so well-controlled] It’s useful also because at least no need to come back every 2 weeks, every 3 weeks, just to titrate the medicines... (S005)  I will try not to [recommend to newly diagnosed patients], because…the rapport is not completely built up, so is not as simple even when you do the tele-consult titration. Sometimes, over the phone we have to talk longer…Not all patients have white coat syndromes. So for those who have it, we do see a better reading at home which is more accurate. So that kind of avoid us from over-prescribing medicine... (S006) | |

Table S8

**Dimension: Workflow and communication / Theme: Communication process**

| **Category** | **Patient quotes** | **Healthcare professional quotes** |  |
| --- | --- | --- | --- |
| Workload management |  | We can allocate our time to see more of the complex patient face-to-face. Whereas not complex [tasks] like titration, can do it over the phone. (S005)  In a way, their time will be reduced a lot, in coming over to polyclinic. Ya, so they have a longer TCU [next follow-up appointment] where halfway in between, we just have to call them, and we can always access their readings…So in a way, overall, the benefit is that patients’ load will be less. Less waiting time in the clinics also… Workload and work scope are enhanced I feel. (S007) | |
| Patient-provider engagement – Feedback and advice | I was not told that if I have irregular BP, someone will call me. So far, I have never received anything, even if I never upload anything…It would be good if there is some feedback…Maybe based on the reading of my BP, someone can just give some advice. To start to get yourself active or you know, your BP is a little bit high or something like that…it’s good to have one dedicated care manager, so at least you are very familiar with this person, this person is very familiar with your condition…(F007)  It would be good that one of the nurses or whoever in the team just calls up the patient, “Do you have any difficulty?”... Of course, when they are in trouble, they will panic, they will call. But what I’m saying is that in between, if it’s a 6-month period, at least once or twice, call them and find out from them any problem with the machine and any problem in taking [BP]... (F122) |  | |
| Patient-provider engagement – Ready support |  | I guess it’s a better relationship with the patient because on and off we will call the patient, like ask how are they, the blood pressure seems to be a little bit high on the system, were you doing it correctly? At the right time? Were you taking the medication as per prescribed? Any lifestyle, diet, exercise changes…So kind of encouraging them. (S005)  I would say it will strengthen the relationship between the patients and doctor…They are more receptive to what we say…They submit the reading, we help to interpret…sometimes we give some compliment… it kind of strengthens the rapport, so they have higher level of trust… easier to talk to them about the management when they come for the subsequent follow-up review. (S006)  Like for the care coordinator, when she handles the BP machine, the gateway, if the patients don’t know how to troubleshoot themselves, they come to see the care coordinator. So these are positive relationships built over time. It’s like making life easier for both of them. (S008) | |
